# Diffuse, Bilateral Prostate Imaging Reporting and Data System (PI-RADS) 3 Changes Reported as Inflammation and Their Relation to Clinically Significant Prostate Cancer: A Retrospective Observational Study

**DOI:** 10.7759/cureus.75101

**Published:** 2024-12-04

**Authors:** Junaid Sofi, Pradip Subedi, Ameena Pradhan

**Affiliations:** 1 Urology, Northwick Park Hospital - London North West University Healthcare NHS Trust, Harrow, GBR

**Keywords:** clinically significant prostate cancer, gleason's score, international society of urologic pathology (isup), mri prostate, pirads 3, prostate cancer (pca)

## Abstract

Aim/Objective

The aim of this study was to investigate if diffuse, bilateral PI-RADS (Prostate Imaging Reporting and Data System) 3 changes, reported on MRI Prostate, harbour clinically significant prostate cancer (csPCa) within them.

Methods

Data from 108 men with diffuse, bilateral PI-RADS 3 changes on MRI of the prostate who underwent systematic prostate biopsy between January 2000 and November 2023 were analyzed. Histology results were classified as benign or malignant, and clinically significant prostate cancer (csPCa) was defined according to the European Association of Urology (EAU) guidelines as ISUP GG (International Society for Urological Pathology Grade Group) 2 or higher. Data were analyzed using SPSS software, version 26.0 (IBM Corp., Armonk, NY, USA).

Results

The analysis showed that 30.5% of men with bilateral diffuse PI-RADS 3 changes had a diagnosis of clinically significant prostate cancer. There was a correlation (p-value < 0.05) of prostate-specific antigen density (PSAd) to the diagnosis of cancer in these diffuse PI-RADS 3 changes.

Conclusion

The likelihood of clinically significant prostate cancer in diffuse PI-RADS 3 changes is quite high, especially when associated with a high PSA density. Therefore, caution is necessary before deciding against biopsying these changes, even if they appear inflammatory on MRI.

## Introduction

The Prostate Imaging Reporting and Data System (PI-RADS) has revolutionized the assessment and management of prostate lesions using multiparametric MRI (mpMRI). In its latest version, PI-RADS v2.1, PI-RADS 3 lesions denote findings that are equivocal, presenting a diagnostic challenge that often requires further evaluation and clinical correlation to determine the appropriate course of action. Currently, there is no consensus among guidelines regarding the management of these lesions [1]. According to the literature, the reported rates of clinically significant prostate cancer in PI-RADS 3 lesions range from 19% to 25% under the PI-RADS v2 classification and 4.5% to 27.2% with PI-RADS v2.1 [2-8]. Most studies conducted after the introduction of PI-RADS v2.1 report an incidence of less than 10% [8]. However, these studies do not distinguish between diffuse and discrete PI-RADS 3 lesions. Diffuse bilateral PI-RADS 3 changes on prostate MRI are commonly described in reports and are often attributed to inflammation or prostatitis. Therefore, we conducted this study to determine the incidence of clinically significant prostate cancer (csPCa) in diffuse PI-RADS 3 changes.

## Materials and methods

We screened suspected prostate cancer referrals, received at Northwick Park Hospital, between January 2020 and November 2023. A total of 108 men met the criteria, which required bilateral diffuse PI-RADS 3 changes on MRI prostate and subsequent history of undergoing a systematic prostate biopsy. These patients were included in the study and data was collected retrospectively. Discrete PI-RADS 3 lesions were excluded from the study. Data were collected on age, ethnicity, digital rectal exam (DRE) findings, family history, prostate volume, prostate-specific antigen (PSA) value, PSA density (PSAd), and histology from the prostate biopsy. Histology was categorized into benign and malignant groups. The benign pathology group included normal prostate tissue, inflammation, high-grade prostatic intraepithelial neoplasm (HGPIN), and atypical small acinar proliferation (ASAP). The malignant group was further divided into clinically insignificant prostate cancer (ciPCa) and clinically significant prostate cancer (csPCa) (Grade Group ≥2, Gleason score ≥3+4) [2]. csPCa was classified using the International Society of Urological Pathologists (ISUP) grade group system [9]. The collected data were analyzed using SPSS v26 (IBM Corp., Armonk, NY, USA). The primary objective was to determine the incidence of csPCa, and the secondary objective was to evaluate whether the detection of csPCa correlated with age, ethnicity, PSAd, abnormal DRE findings, and family history. Categorical data were analyzed using Fisher's exact test, and continuous data were analyzed using an independent t-test.

## Results

Patient characteristics

Characteristics of patients (n=108) considered for analysis were collected (Table 1). Ethnicity was classified according to the Office of National Statistics classification [10].

**Table 1:** Patient characteristics PSA: Prostate-specific antigen, PSAd: Prostate-specific antigen density, DRE: Digital rectal examination.

Patient characteristics	Values	
Age (years)	Mean	61.15±7.21
	Range	41-78
PSA (ug/L)	Mean	6.8±4.97
	Range	0.37-34.70
PSAd (ng/mL^2^)	Mean	0.22±0.19
	Range	0.02-1.2
Family History	Present	22
	Absent	86
Abnormal DRE	Yes	4
	No	104
Ethnicity	Asian or Asian British	29
	Black, Black British, Caribbean or African	34
	Mixed or multiple ethnic groups	06
	White	35
	Other ethnic group	04

Histology

Out of 108 men, 33 men (30.5%) were diagnosed with csPCa, while 15 men (13.8%) were diagnosed with ciPCa and 60 men had benign diagnoses: 42 (38.8%) with normal prostate tissue, 9 (8.3%) with inflammation, 2 (1.8%) with HGPIN and 7 (6.4%) with ASAP (Table 2).

**Table 2:** Distribution of histology across the diffuse bilateral PI-RADS changes HGPIN: High-grade prostatic intraepithelial neoplasm, ASAP: Atypical small acinar proliferation, GG: International Society of Urological Pathology Grade Group

Histology	Benign diagnoses and clinically insignificant cancer					Clinically significant cancer			Total
	Normal	Inflammation	HGPIN	ASAP	GG1	GG2	GG3	GG4	
	42	9	2	7	15	21	7	5	108
Total	75					33 (30.5%)			

Data were divided into ciPCa plus benign diagnosis and csPCa and further analysed. Among the variables compared between the groups, there was no statistical difference in terms of age, PSA, abnormal DRE findings and positive family history. However, there was a statistically significant difference in PSA density between the two groups, with PSAd being higher in the group found to have csPCa. Twenty-two out of 108 men had a family history of prostate cancer. Among men with positive family history, four were diagnosed with csPC, six with ciPC and 12 had benign diagnosis. There was no significant impact of family history on the incidence of csPCa in these diffuse PI-RADS3 changes, although the sample size is too small to draw any conclusions. Among men with abnormal DRE, histology was benign in all (Table 3).

**Table 3:** Comparison of demographic, clinical and biochemical variables between the groups c: p-value<0.05, PSA: Prostate-specific antigen, PSAd: Prostate-specific antigen density, DRE: Digital rectal examination.

Characteristics	Clinically insignificant prostate cancer and benign diagnoses (n=75)	Clinically significant prostate cancer (n=33)	p-value
Age	60.41±7.74	62.82±5.57	0.072
PSA (ug/L)	6.55±5.27	7.47±4.23	0.340
PSAd (ng/mL^2^)	0.18±0.15	0.31±0.23	0.008^c^
Abnormal DRE findings	4 (4/75)	0 (0/33)	0.311
Positive Family history	18 (18/75)	4 (4/33)	0.158

## Discussion

The PI-RADS system classifies prostate lesions on a Likert-like scoring scale of 1-5 based on anatomical characterization using T2 weighted images and functional characterization using DWI (diffusion-weighted imaging) and DCE (dynamic contrast-enhanced imaging). PI-RADS 3 lesions are considered equivocal, unlike PI-RADS 1-2 or PI-RADS 4-5 lesions, and their interpretation is subject to interobserver variability [1]. Diffuse PI-RADS 3 changes on MRI are frequently reported as inflammation or prostatitis. Prostatitis on MRI typically appears as streaky or wedge-shaped decreases in T2W signals along the prostatic septa in the peripheral zone (PZ), with heightened contrast enhancement, increased DWI signals, and a mild to moderate reduction in the apparent diffusion coefficient (ADC). However, in more severe cases, these changes may appear diffuse or patchy, complicating differentiation from clinically significant cancer [11].

In such cases, widespread contrast enhancement associated with extensive prostatitis can obscure the focal enhancement of lesions on DCE imaging, making it difficult to distinguish PI-RADS 3 from PI-RADS 4 lesions in the PZ. The extent of ADC reduction can assist in differentiating clinically significant prostate cancer, which tends to have lower ADC values, from prostatitis, which typically has higher ADC values. However, there is considerable overlap [12]. Merat et al. highlighted that mean ADC is the best marker for differentiating between ciPCa and csPCa in the presence of inflammation, reporting a cancer detection rate of only 7.7% among 52 diffuse PI-RADS 3 lesions [13]. Rourke et al. identified inflammation as a common cause of false-positive MRI findings, leading to negative biopsies [14]. Liddell et al. reported an overall csPCa detection rate of 6.5% in 93 PI-RADS 3 lesions, with 10.8% in the peripheral zone (PZ) and 3.8% in the transitional zone (TZ) [7]. Similarly, Ahmed et al. found a csPCa detection rate of 20.8% among 163 PI-RADS 3 lesions [2].

These findings suggest variability in csPCa detection in PI-RADS 3 lesions, partly due to the failure to distinguish between discrete and diffuse lesions and the under-biopsy of diffuse PI-RADS 3 changes. Our study focuses exclusively on diffuse bilateral PI-RADS 3 changes, reporting a csPCa detection rate of 30.5%, which is higher than previous studies. This higher detection rate may reflect the inclusion of only diffuse bilateral PI-RADS 3 changes, emphasizing the potential contribution of inflammation to prostate cancer aetiology.

The limitations of our study include its retrospective design and small sample size. Additionally, the study cohort was restricted to bilateral and diffuse PI-RADS 3 changes, representing a specific subset, which may limit the generalizability of our findings to all PI-RADS 3 lesions.

## Conclusions

This study demonstrates a significant incidence of clinically significant prostate cancer in men with diffuse, bilateral PI-RADS 3 changes, which are reported as prostatitis or inflammation on MRI. Caution is necessary before deciding not to biopsy the prostate in these cases. Although the number of bilateral PI-RADS 3 changes suggestive of inflammation may be small compared to the large number of prostate biopsies performed annually for other target lesions, missing a cancer diagnosis by not considering these lesions for biopsy could be catastrophic.
